# Urine-based antigen detection assay for diagnosis of visceral leishmaniasis using monoclonal antibodies specific for six protein biomarkers of *Leishmania infantum / Leishmania donovani*

**DOI:** 10.1371/journal.pntd.0008246

**Published:** 2020-04-23

**Authors:** Claudia Abeijon, Fabiana Alves, Séverine Monnerat, Jane Mbui, Agostinho G. Viana, Raquel M. Almeida, Lilian L. Bueno, Ricardo T. Fujiwara, Antonio Campos-Neto

**Affiliations:** 1 DetectoGen Inc., Grafton, Massachusetts, United States of America; 2 Drugs for Neglected Diseases *Initiative*, Geneva, Switzerland; 3 KEMRI Centre for Clinical Research, Nairobi, Kenya; 4 Federal University of Minas Gerais, Belo Horizonte, MG, Brazil; 5 Cummings School of Veterinary Medicine at Tufts University, North Grafton, Massachusetts, United States of America; Instituto Goncalo Moniz-FIOCRUZ, BRAZIL

## Abstract

The development of an accurate protein-based antigen detection assay for diagnosis of active visceral leishmaniasis (VL) would represent a major clinical advance. VL is a serious and fatal disease caused by the parasites *Leishmania infantum* and *Leishmania donovani*. The gold standard confirmatory diagnostic test for VL is the demonstration of parasites or their DNA from aspirates from spleen, lymph node, and bone marrow or from blood buffy coats. Here we describe the production and use of monoclonal antibodies (mAbs) for the development of a sensitive and specific antigen detection capture ELISA for VL diagnosis. This test simultaneously detects six leishmania protein biomarkers that we have previously described (*Li-isd1*, *Li-txn1*, *Li-ntf2*, *Ld-mao1*, *Ld-ppi1* and *Ld-mad1*). The initial clinical validation of this new mAb-based multiplexed capture ELISA showed a sensitivity of ≥93%. The test was negative with 35 urine samples from healthy control subjects as well as with 30 patients with confirmed non-VL tropical diseases (cutaneous leishmaniasis, n = 6; Chagas disease, n = 6; schistosomiasis, n = 6; and tuberculosis, n = 12). These results strongly support the possible utility of this mAb-based multiplexed capture ELISA as a promising diagnostic test for active VL as well as for monitoring the treatment efficacy of this disease. The test is ready for upscaling and validation for clinical use.

## Author summary

Visceral leishmaniasis (VL) or kala-azar is a systemic parasitic disease that is endemic in 75 countries with more than 500 million people at risk of infection. VL is caused primarily by the parasites known as *Leishmania infantum* and *Leishmania donovani*. It is estimated that VL affects 50,000–90,000 people each year, with 90% of the cases occurring in India, Kenya, Somalia, Sudan, South Sudan and Brazil. VL is a fatal disease if not treated. Laboratory diagnosis of this disease can be performed using several different approaches. However, there is still a need for a simple, non-expensive, sensitive and specific test that can be used not only for the accurate diagnosis of this serious disease but also to monitor the efficacy of its treatment. Here we describe a new test that can circumvent most of the drawbacks of existing approaches. This new test detects six leishmanial proteins or biomarkers that are eliminated in the urine of patients with VL. The test is a conventional ELISA assembled with monoclonal antibodies (mAbs) specific for each of the six different biomarkers and it is performed in urine samples of suspected patients. An initial clinical validation demonstrated that the test has a sensitivity of ≥93% and specificity of 100%. This new and accurate mAb-based multiplexed assay could be a useful resource to diagnose most clinical forms and/or the severity of the disease e.g., active VL, asymptomatic VL, post kala-azar dermal leishmaniasis, VL/HIV co-infection, etc.

## Introduction

Visceral leishmaniasis (VL) is a serious parasitic disease caused by *Leishmania donovani / Leishmania infantum* [1, 2]. Diagnosis of VL is routinely performed by invasive splenic, bone marrow, or lymph node aspiration, followed by microscopic identification of the parasites and or culture. The sensitivity of these tests is in general modest and varies enormously. Nucleic acid amplification tests have better sensitivity [3] but are relatively more complicated, expensive, and are restricted to referral hospitals and advanced research centers [4–12]. Due to the limitations of these invasive approaches, the presence of anti-parasite antibodies in serum is also routinely used to diagnose VL [13, 14]. However, antibody tests have variable sensitivity in different endemic regions [15–17], and cannot discriminate active disease from cured individuals. An antigen detection test that detects parasite carbohydrate antigens in urine of VL patients with active disease was developed several years ago [18–21]. Unfortunately, the sensitivity/specificity of the test varied widely, probably due to the heterogeneity of the parasites’ carbohydrate antigens.

We have recently developed an alternative approach to circumvent these restrictions: a multiplexed capture ELISA that detects the *L*. *donovani* / *L*. *infantum* protein biomarkers *Li-isd1*, *Li-txn1*, *Li-ntf2*, *Ld-mao1* and *Ld-ppi1* [22]. These proteins were previously discovered using mass spectroscopy in the urine of VL patients [23–25]. The multiplexed assay was assembled with polyclonal rabbit IgG and chicken IgY antibodies specific for these five antigens and showed a sensitivity of 82.2% for the diagnosis of VL. A sixth biomarker (*Ld-mad1*), which was also initially discovered, was difficult to express as a recombinant protein and was not evaluated in these former studies. Although polyclonal antibody-based tests (in contrast to monoclonal antibodies) are not ideal for upscaling and commercial production, these proof-of concept and preliminary results validated the utility of the discovered leishmanial protein biomarkers found in urine of VL patients as powerful tools for the development of an accurate diagnostic test for this disease.

Here we report the development of a monoclonal antibody-based multiplexed capture ELISA that detects the six VL protein biomarkers previously described. The initial clinical validation of this new VL multiplexed test showed a sensitivity of ≥93% and a specificity of 100%.

## Material and methods

### Clinical specimens

A total of 24 urine samples of VL patients from Brazil, 12 females and 12 males, ages 2-65y, assumed to be infected with *L*. *infantum*, and 45 urine samples of VL patients from Kenya, 18 females and 27 males, ages 6-36y assumed to be infected with *L*. *donovani*, were evaluated in this study. These samples were collected before the initiation of therapy and were from patients diagnosed with VL based on the following criteria: a clinical course consistent with VL (e.g., fever, anemia, hepatosplenomegaly), and confirmatory laboratory findings (identification of *Leishmania* in spleen or bone marrow aspirates) and positive serological test. None of the patients had any clinical symptoms or laboratory findings compatible with renal or urinary tract abnormalities, nor were any of them receiving anti *Leishmania* therapy at the time of urine collection. In addition, none of the VL patiens were positive for HIV.

### Ethics statement

**Fig 1:**
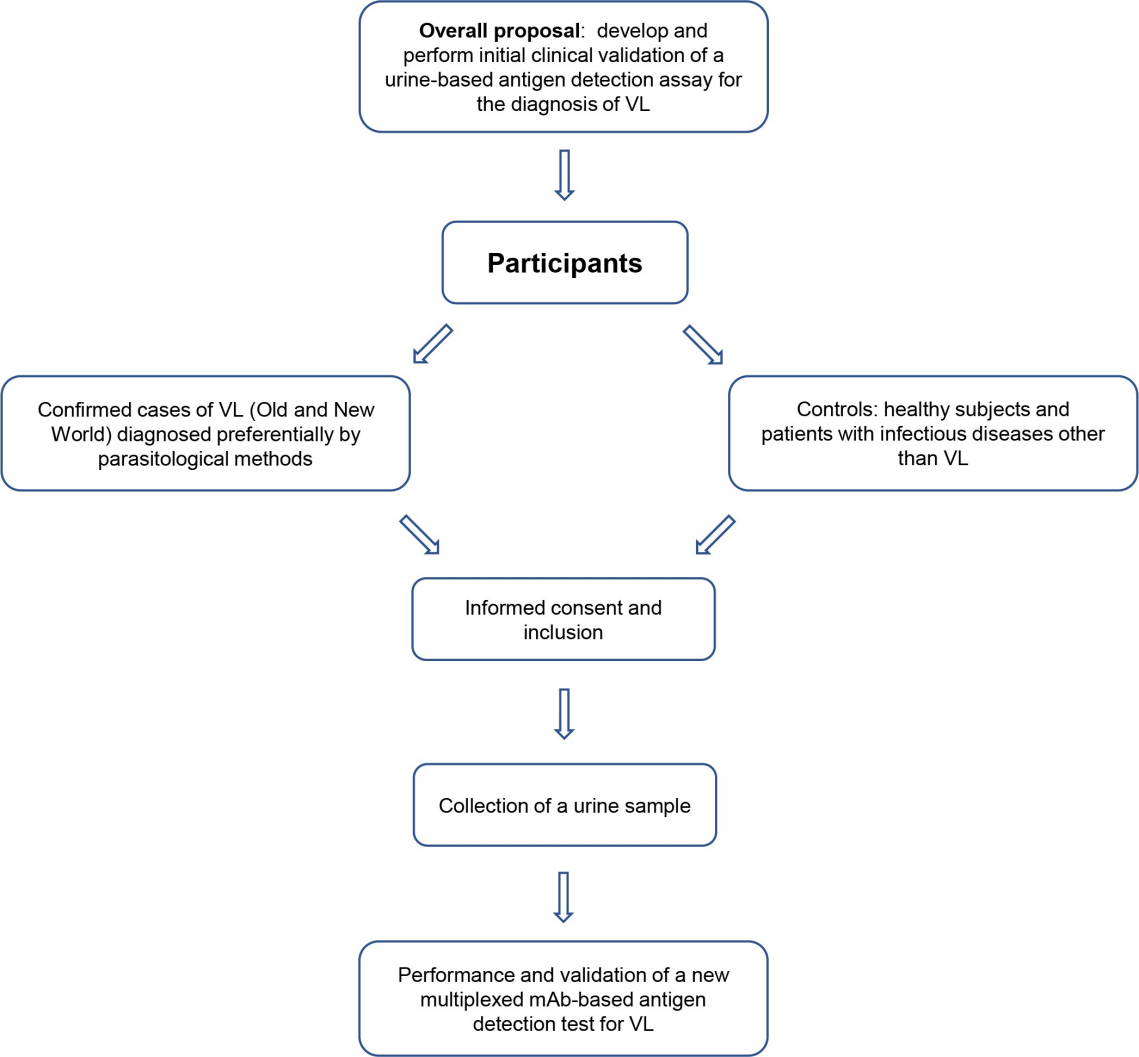
Diagram of data analysis plan.

All samples from Brazil (VL patients and controls) were obtained from the University Hospital Clemente Farias (Montes Claros, Minas Gerais, Brazil). Clearance approval to use these samples was obtained from the Human Research Ethics Committee—COEP (CAAE -00842112.2.0000.5149) of the Federal University of Minas Gerais. The samples from Kenya were obtained from Kacheliba County Hospital (West Pokot County) and from Kimalel Health Center (Baringo County). Clearance approval to use these samples was obtained from the KEMRI Scientific and Ethics Review Unit (KEMRI/SERU/CCR/0011/3120). The control samples included 35 urine samples obtained from healthy control subjects living in the same geographical areas as the VL patients. In addition, control samples from non-VL patients from Brazil who had other infectious diseases (cutaneous leishmaniasis, n = 6; Chagas disease, n = 6; schistosomiasis, n = 6; and tuberculosis, n = 12) were also included. The serological tests for VL were negative in all control samples. All samples used in this study were anonymized. The overall data analysis plan for the study is illustrated in Fig 1.

### Leishmania donovani *protein expression and purification*

The DNA sequence coding for the *L*. *donovani Ld-mad1* was codon optimized for expression in *E*. *coli*. The gene was synthesized by Blue Heron (Bothell, WA). To allow sub-cloning, restriction enzyme sequences Nde I and Bam HI were included at the 5’ and 3’ endings, respectively, of the optimized DNA fragment. The synthetic gene was digested with the restriction enzymes and sub-cloned into a pET-14b expression vector, which was similarly digested for directional cloning. Protein expressed in the pET-14b expression vector contains a six-residue histidine tag at the N-terminus of the molecule, which facilitates purification by affinity on QIAexpress Ni-NTA agarose matrix (Qiagen, Valencia, CA). Recombinant protein was obtained and purified from 100ml of IPTG-induced batch cultures by affinity chromatography using QIAexpress Ni-NTA agarose matrix (QIAGEN, Chatsworth, CA) as described [26]. The yields of recombinant proteins were 10-20mg per liter of induced bacterial culture, and purity was assessed by SDS-PAGE followed by Coomassie blue staining.

### Generation of specific monoclonal antibodies (mAbs)

The generation and purification of the mAbs were outsourced to GenScript USA Inc, Piscataway, NJ 08854 USA and followed standard procedures [27, 28]. Briefly, the purified recombinant proteins *Li-isd1*, *Li-txn1*, *Li-ntf2*, *Ld-mao1*, *Ld-ppi1* and *Ld-mad1* (50μg of each) were individually emulsified with an equal volume of complete Freund’s adjuvant and injected subcutaneously into three C57BL/6 mice per antigen. The animals were given two subcutaneous boosters (25μg of protein in IFA) two weeks apart. One week after the first boost the animals were bled and serum was collected and tested by ELISA to determine the titer of each antiserum. The mouse producing the highest titer of IgG specific for each marker was selected for production of the mAbs. The mice were sacrificed three days after the second boost, their spleens were harvested and the spleen cells were fused with the mieloma cell line SP2/0 for generation of hybridomas. Monoclonal hybridoma clones were then obtained by limiting dilution and their supernatants were tested for the presence of specific IgG antibody using both quantitative ELISA and Western blot analysis. Twenty clones were selected for each marker. IgG mAbs were purified from the hybridoma supernatants by affinity chromatography using recombinant protein A/G immobilized resin [29]. Aliquots of selected IgG mAbs were subsequently biotin labeled [30].

### Epitope recognition by mAbs

Supernatants from each of the 20 hybridoma clones were tested for their reactivity with synthetic purified 20mer peptides covering the entire full length of each biomarker and overlapping by 10 amino acids. Reactivity was tested by direct ELISA as previously described [31]. Peptides were synthesized by GenScript (Piscataway, NJ).

### Western Blot

Purified recombinant *Ld-mad1* (50 ng) and whole lysate extract from *L*. *donovani* amastigotes and promastigotes were fractionated by SDS-PAGE (4–20% gradient gel) and transferred to polyvinylidene fluoride membrane (PVDF, Millipore, Medford, MA). Whole lysate of *L*. *donovani* from promastigote parasites was prepared from the organisms cultured for 7–10 days in complete Schneider’s medium at 26°C. Whole lysate of *L*. *donovani* amastigotes was from *in vitro* infected macrophage cell line DH82 [32]. Briefly, macrophages were grown in RPMI medium supplemented with 10% fetal calf serum followed by exposure to *L*. *donovani* promastigotes at a ratio of 50/1 (parasites/macrophages). The infection was performed for 24 hours at 37°C and with 5% CO_2_. Non-internalized promastigotes were then removed by washing and infection continued for an additional 48 hours. Infected cells were then removed after addition of EDTA/trypsin (three minutes) followed by centrifugation at 1000g for 10 minutes. Pellets were then disrupted with electrophoresis loading buffer and used in Western blot analysis. The blots were blocked overnight at 4°C with tris-buffered saline with 0.1% Tween 20 (TBS-T) containing 1% bovine serum albumin (BSA) and subsequently probed with a pool of biotinylated monoclonal IgG antibodies specific for *Ld-mad1*. After several rinses with TBS-T, streptavidin labeled with horseradish peroxidase (Thermo Scientific Pierce, Rockford, IL) was added. After additional washings, bound conjugates were detected using the ECL enhanced chemiluminescence system (Amersham/GE Healthcare, Piscataway, NJ) and proteins were visualized on a ChimiDocTouch Imaging System from BioRad, Hercules, CA.

### ELISA

A capture ELISA antigen detection test was developed using purified mAb specific for different epitopes in each leishmanial biomarker. Briefly, 96 well ELISA plates (EIA/RIA plate High Binding, Corning International, Corning, NY) were coated overnight at 4°C with mAbs in PBS at a concentration of 2ug/ml. The multiplexed ELISA, designed to detect simultaneously *Li-isd1*, *Li-txn1*, *and Li-ntf2*, *Ld-mao1*, *Ld-ppi1*, and *Ld-mad1* was assembled using as capture reagent a pool of the six specific mAbs (mAbP11, mAbP212, mAbP33, mAbP41, mAbP64 and mAbP713) at 2ug/ml each. Wells were washed with PBS+ 0.1% Tween-20 (Sigma Chemical Co., St. Luis, MO) and blocked at room temperature with PBS + 1% BSA + 0.1% Tween 20 (PBS/BSA/Tween) for 2h. After washing, recombinant antigen or human urine samples were added and incubated overnight at 4°C. Plates were washed, followed by incubation for 1h with a biotin labeled mAb that recognizes a different epitope than that recognized by the mAb that was used for capture. For the multiplex assay, the developing reagent consisted of a pool of the biotinylated mAbs used in the individual assays (mAbP115, mAbP218, mAbP311, mAbP49, mAbP618, and mAbP79). Following several rinses in PBS/BSA/Tween, peroxidase-labeled streptavidin at 1:2,000 dilution (BD Bioscience, Franklin Lakes, NJ) was added for 30 minutes. The plates were then washed and reactions were developed with TMB Microwell Peroxidase substrate system (KPL, Gaithersburg, MD 20878) and read at 450 nm.

## Results

### Leishmanial protein biomarkers present in urine of VL patients

In recent years we have discovered and described several *L*. *infantum* and *L*. *donovani* proteins excreted in the urine of patients with VL from Brazil, Kenya and India [22–25]. Six of them have been selected as targets for the development of a diagnostic antigen detection assay. The selection criteria for these peptides included: a high MS XCorr >3.0; BLAST analysis of the peptide matching the pathogen’s biomarker having a highly significant E-value; finding two or more peptides per donor protein; found in urine samples from at least two out of ten patients; and ideally with a MW ≤30–40 kDa. Table 1 describes these selected biomarkers, which were previously discovered in the urine of VL patients from Brazil, Kenya and India [22–25]. As can be seen, three of the six biomarkers were found in the urine of VL patients from these three countries and three were found only in urine of patients from Kenya and India. With the exception of *Ld-mad1*, we have tested the possible utility of these biomarkers for the initial development of a proof of concept urine-based antigen detection assay for the diagnosis of VL. We produced and purified pairs of polyclonal antibodies (rabbit IgG and chicken IgY) specific for each of these biomarkers and used them to assemble capture ELISA to detect them in the urine of VL patients and controls. A multiplexed assay assembled for the detection of *Li-isd1*, *Li-txn1 and Li-ntf2* originally discovered in urine samples of VL patients from Brazil (where the disease is caused by *L*. *infantum*) proved to be highly sensitive and specific for the diagnosis of VL occurring in patients from this country (~95% and 100% respectively). In contrast, the test was less accurate for the diagnosis of VL occurring in patients from Kenya and India, where the disease is caused by *L*. *donovani*. However, the inclusion of *Ld-mao1* and *Ld-ppi1* (previously discovered in the urine of VL patients from Kenya and India) improved the sensitivity of the test to nearly clinically acceptable levels of 82% [22].

**Table 1:** *Leishmania* peptides previously identified by mass spectroscopy in individual urine samples of patients with VL from Brazil, Kenya and India.

Leishmanial donor protein of discovered peptide (putative)	Accession	Peptide identified in patient urine	MW of donor protein	Frequency in urine samples of patients from:		
				Brazil	Kenya	India
Iron superoxide dismutase (*Li-isd1*)	XM_001467829	K.LNAAAESNSGLASK.S R.GGGEPSGPLASAIVDSFGSFASFK.K	21.53	1/5	5/6	1/9
Tryparedoxin (*Li-txn1*)	XM_001466605	K.HLGDVLK.L K.QNDMVDMSSLSGK.T K.LVEFYEK.H K.MPWLSIPFEK.R R.NVVEALTK.Q K.QYKVESIPTLIGLNADTGDTVTTR.A R.HALTQDPEGEQFPWRDE.-	16.7	2/5	5/6	5/9
Nuclear transport factor 2 (*Li-ntf2*)	XM_001463701	R.DQLAGIYRPNTLLTWQK.E K.EQVQGVDAIMAR.F R.FANLGFTEAAFK.Q	13.89	2/5	4/6	4/9
Maoc family dehydratase *(Ld-mao1)*	XP_003858460.1	K.EMPGPGTVYLSQNLR.F K.GLISLSNIVQK.T K.TLQFEGESEWSVTK	16.97	Not found	2/6	2/9
Peptidyl-prolyl Isomerase *(Ld-ppi1)*	XP_003858557.1	R.RTGQPTTISYEEAVTELQK.W R.VTFEEAAR.Q	12.62	Not found	2/6	1/9
Malate dehydrogenase *(Ld-mad1)*	XP_003864180.1	R.VAVLGAAGGIGQPLSLLLK.N R.DDLFNTNASIVR.D R.LFGVTTLDVVR.A R.IQFGGDEVVK.A	33.28	Not found	3/6	6/9

**Fig 2:**
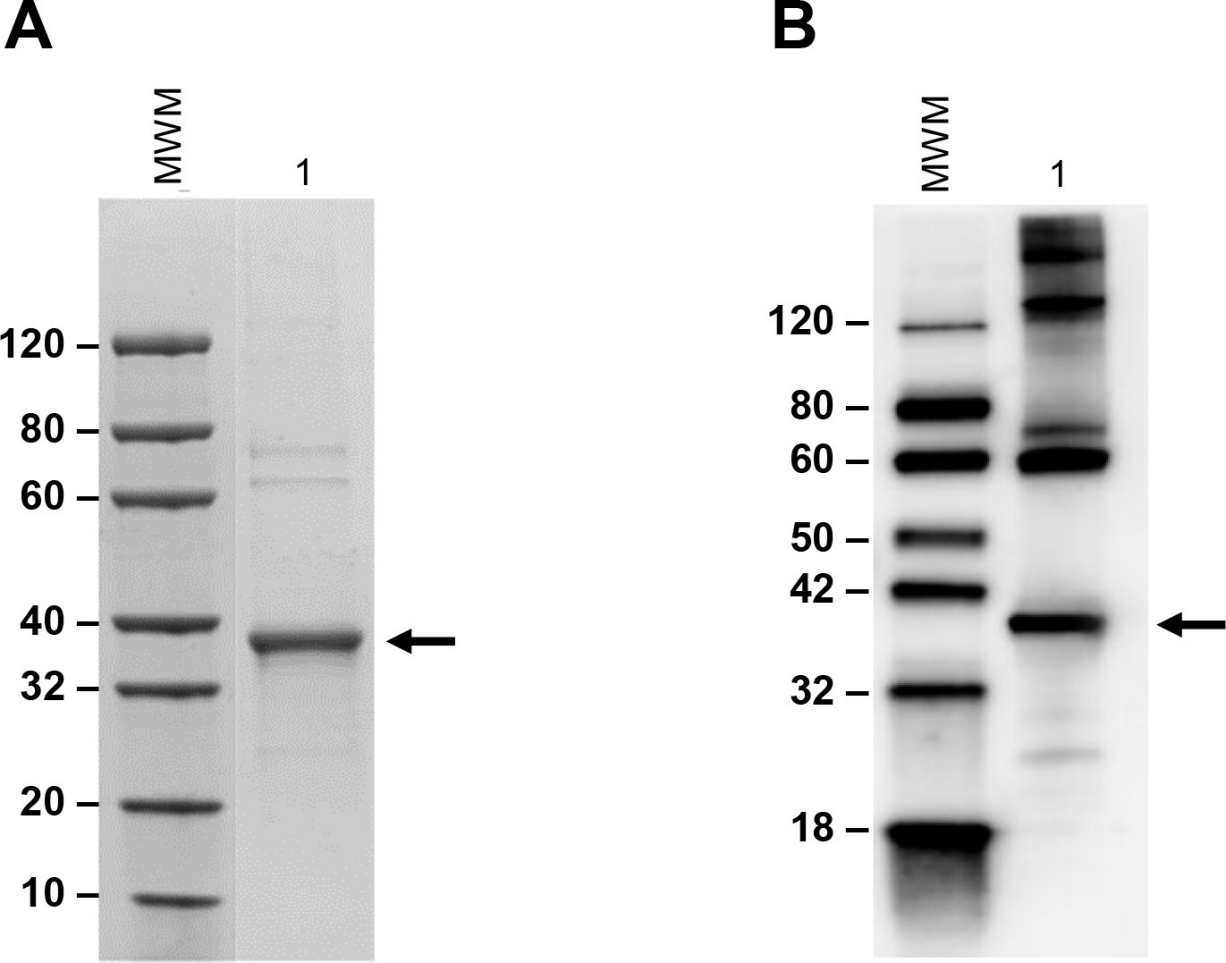
**SDS-PAGE (A) and Western blot (B) analysis of purified recombinant *Ld-mad1*.** Recombinant protein containing His-tag amino terminal residues were expressed in *E*. *coli* BL21(DE3)pLysS followed by purification by affinity chromatography using Ni-NTA agarose matrix. Purity was evaluated by SDS-PAGE (4–20% gradient polyacrylamide gel) Coomassie blue staining (A) and by Western blot using anti-His-tag mAb (B). Arrows point to a protein band with MW that matches the deduced MW of *Ld-mad1*. Several other bands of higher MW are seen in the Western blot; since these bands are revealed with an anti-His tag mAb they are possibly aggregates or polymers of *Ld-mad1*.

Unfortunately, at that time we were unable to assemble a multiplexed test that included *Ld-mad1*, which is a biomarker that was present in several urine samples from VL patients from Kenya and India (Table 1). In order to include this biomarker in the multiplexed assay, we produced and purified the recombinant molecule to be used for the generation of specific antibodies. Before immunization, the purity of the recombinant *Ld-mad1* was assessed and confirmed by SDS-PAGE with Coomassie blue staining and Western blot analysis (Fig 2).

**Fig 3:**
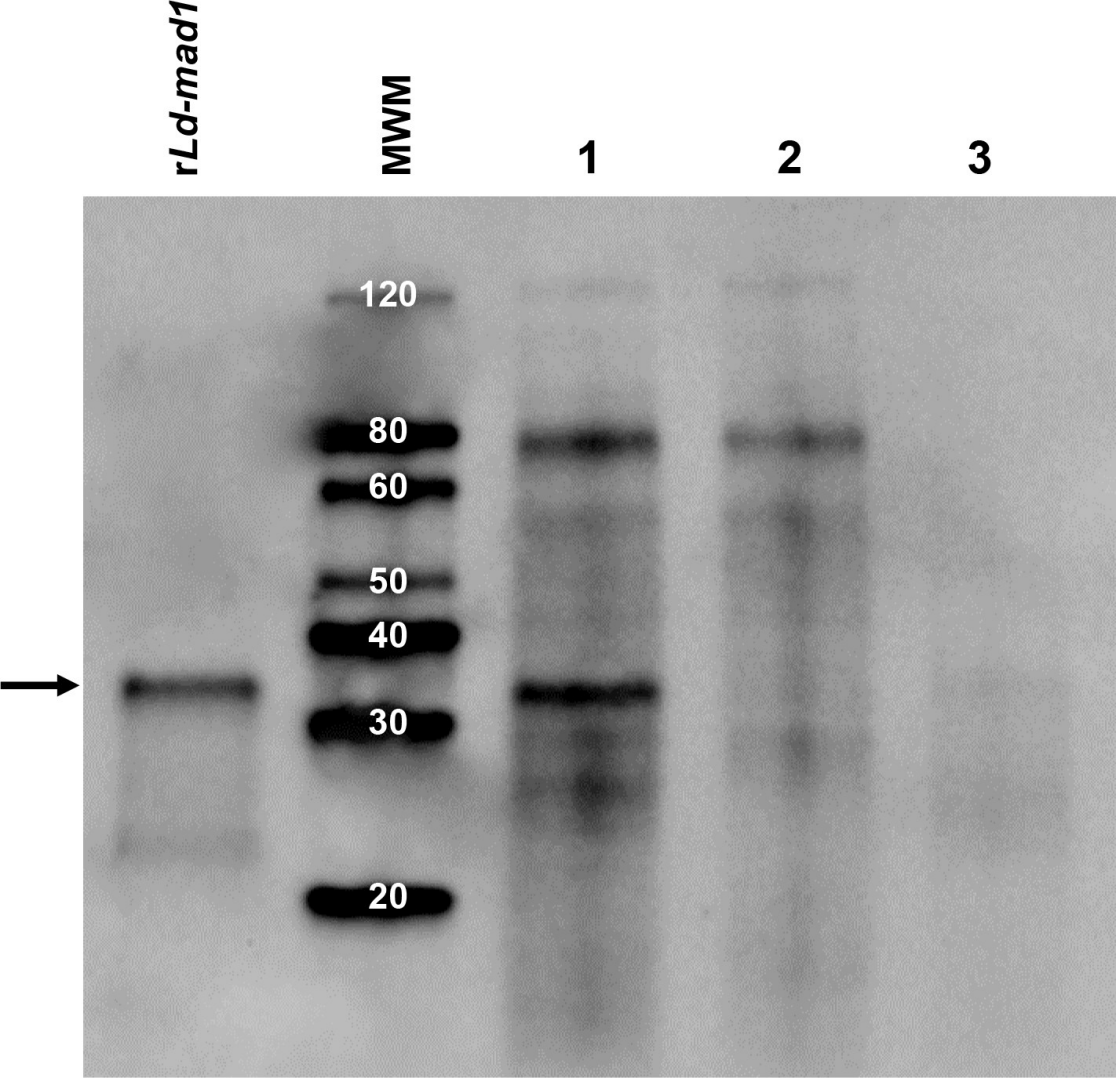
Immunochemical detection of *Ld-mad1* in *L*. *donovani* amastigotes. Validation of *Ld-mad1* as a genuine protein produced by *L*. *donovani* was performed by Western blot using amastigote and promastigote cell lysates from the parasite. *L*. *donovani*-infected macrophage DH82 was used as the source of the amastigote forms of the parasite. The antibody was a pool of three mAbs specific for *Ld-mad1*. *rLd-mad1*, purified recombinant protein; MWM, Markers and respective MWs; Lane 1, crude extract of macrophage DH82 infected with *L*. *donovani*; Lane 2, crude extract of non-infected macrophage DH82; Lane 3, crude extract of *L*. *donovani* promastigotes. The black arrow points to *Ld-mad1* recombinant protein band recognized by the mAbs. A protein band of the same MW as *Ld-mad1* that is present only in the cell lysate of *L*. *donovani* infected macrophages. No bands that correspond to the MW of *Ld-mad1* were seen in membranes blotted with the same amastigote and promastigote cell lysates and probed with an irrelevant mAb.

Western blot analysis using specific monoclonal antibodies (below) and whole cell extract from both *L. donovani* promastigotes and amastigotes confirmed that the purified *Ld-mad1* protein matches a genuine leishmanial molecule. Both stages of the parasite cell cycle were included because previous publications suggested that *Ld-mad1* is primarily expressed by amastigotes [33, 34]. Western blot analysis confirmed that the native *Ld-mad1* protein is barely detected in promastigote forms of the parasite and is clearly expressed by amastigotes (Fig 3). Importantly, these results validate that the recombinant *Ld-mad1* protein is an accurate copy of the native molecule produced by *L*. *donovani*.

### Production and characterization of monoclonal antibodies specific for the leishmanial biomarkers *Li-isd1*, *Li-txn1*, *Li-ntf2*, *Ld-mao1*, *Ld-ppi1 and Ld-mad1*

**Fig 4:**
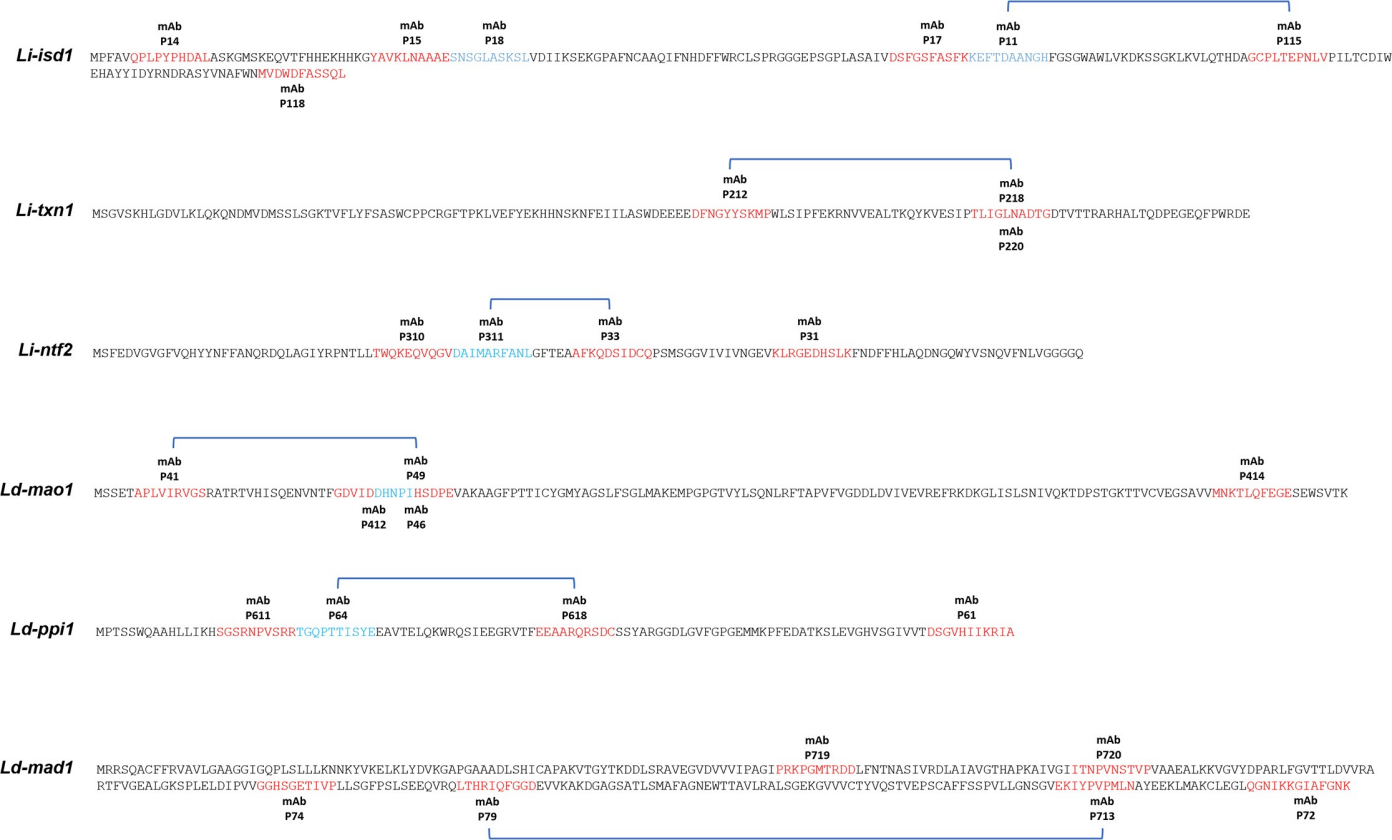
Mapping of epitope recognition by IgG mAbs specific to the leishmanial biomarkers *Li-isd1*, *Li-txn1*, *Li-ntf2*, *Ld-mao1*, *Ld-ppi1 and Ld-mad1*. IgG mAbs were purified from supernatants of cloned hybridoma cells that recognized different peptides on the leishmanial biomarkers and were subsequently biotin labeled. Mapping of epitope recognition was performed by direct ELISA using plates coated with synthetic purified 20mer peptides covering the entire full length of each biomarker and overlapping by 10 amino acids. Letters in red and blue represent a sequence of 10–15 amino acids or the epitopes that were recognized by the indicated mAbs. Blue brackets illustrate the pair of antibodies that were selected as the capture and detecting reagents of the assembled capture ELISAs.

Subsequent to immunization of mice and generation of hybridomas, we performed limiting dilution followed by determination of their reactivity with the six leishmanial biomarkers, which was tested by direct ELISA and Western blot. For each biomarker we selected 20 IgG positive hybridoma clones for mapping of epitope recognition. This step aimed to select clones producing mAbs that would recognize different epitopes in each protein, which are essential for the assembling of a capture ELISA to detect the markers. The epitope mapping was performed by direct ELISA using synthetic 20mer peptides covering the entire full length of each biomarker and overlapping by 10 amino acids. This protocol allowed the detection of several mAbs (Table 2) that were specific for different epitopes spanning from the N to the C terminus of each biomarker. Several supernatants from the hybridoma clones did not clearly recognize the 20mer peptides. Fig 4 depicts the epitope recognition of the mAbs that recognized the 20mer peptides. The mAbs were designated by the prefix P1, P2, P3, P4, P6, P7 (for *Li-isd1*, *Li-txn1*, *Li-ntf2*, *Ld-mao1*, *Ld-ppi1 and Ld-mad1* respectively) followed by a number that corresponds to the hybridoma clones labeled from 1–20. The hybridoma clones that are listed in Fig 4 were then expanded for production and purification of the IgG mAbs. Approximately 10–15 mg of purified IgG were obtained for each clone. From each purified mAb, an aliquot of 2 mg was separated and biotinylated.

**Table 2:** Number of generated hybridoma clones producing mAbs specific for different epitopes in each leishmanial biomarker.

Biomarker	# of epitopes recognized by different mAbs
*Li-isd1*	7
*Li-txn1*	3
*Li-ntf2*	4
*Ld-mao1*	5
*Ld-ppi1*	4
*Ld-mad1*	6

**Fig 5:**
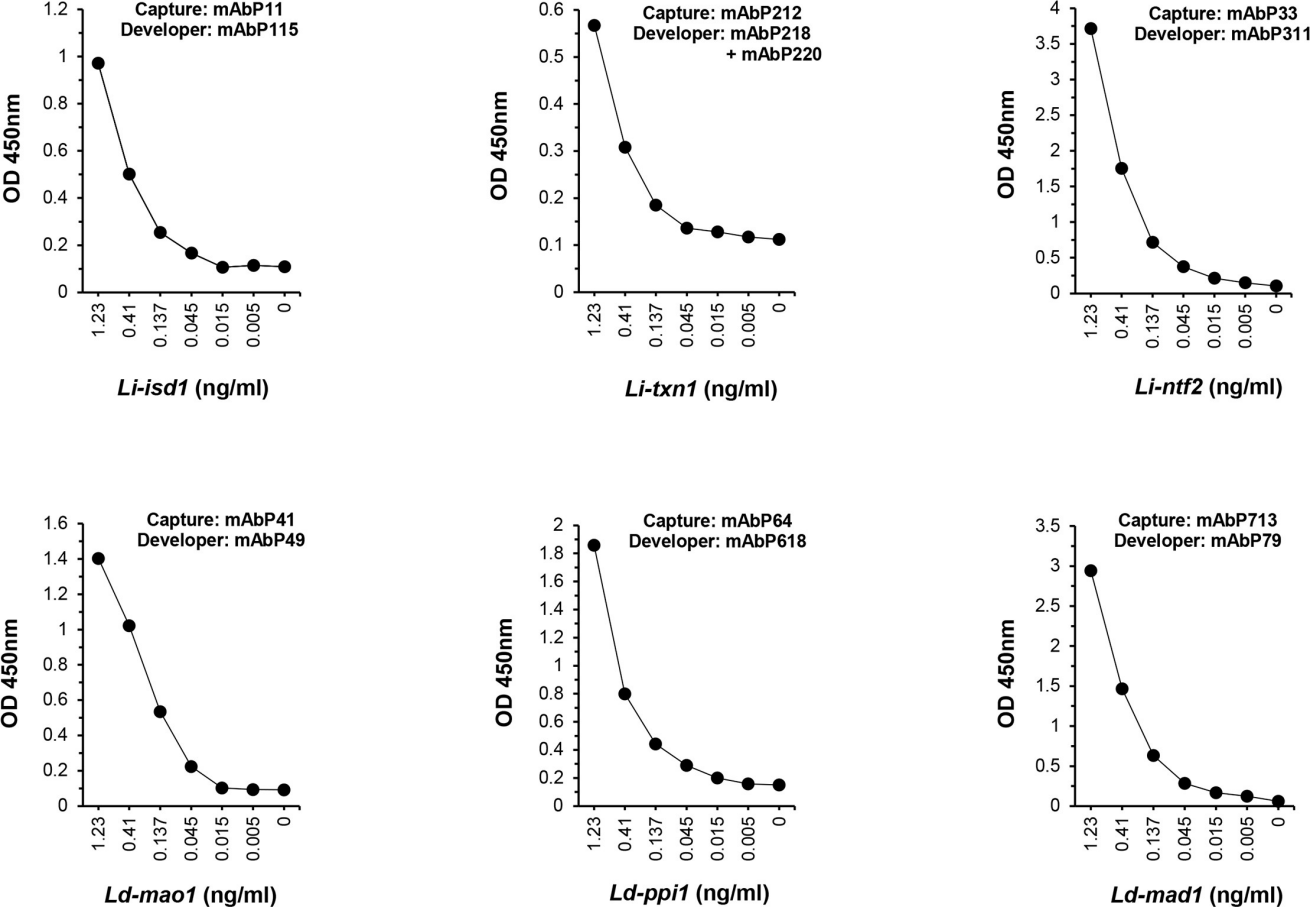
Determination of the limit of detection (LOD) of capture ELISAs assembled to individually detect the protein biomarkers *Li-isd1*, *Li-txn1*, *Li-ntf2*, *Ld-mao1*, *Ld-ppi1 and Ld-mad1* spiked in urine samples of normal healthy subjects. Capture mAbs at previously determined concentration of 200 ng/well were used to coat the ELISA plates that were prepare to detect each biomarker individually. Wells were then incubated with various concentrations of the antigen diluted in urine from normal healthy subjects followed by incubation with biotin labeled developer mAb (2 μg/ml). Reactions were developed after addition of streptavidin labeled peroxidase, substrate (H_2_O_2_) and the chromophore TMB. Results are expressed as OD read at 450nm. Note that for all six assays the LOD was ≤45 pg/ml of biomarker.

To select the mAb producing hybridoma clones for the development of the final capture ELISA, each purified IgG mAb was tested either as a capture or developing reagent using a checkerboard approach, i.e., each of all mAbs specific for one epitope of one of the biomarkers was tested as a capture reagent, paired with all other purified biotinylated mAbs specific for the other epitopes of the same biomarker. Fig 5 illustrates the pairs of antibodies that provided the best signal to noise results for each biomarker. The criterion used for the selection of the best pairs of antibodies was primarily those combinations that would yield a sensitivity or limiting of dilution that was equal to or smaller than 45 pg/ml in a capture ELISA performed with urine spiked with the biomarker. Interestingly and surprisingly, the best combination of mAb pairs comprised of antibodies that recognize epitopes that are almost contiguous to each other, in opposition to epitopes that are located far from each other in the protein molecule (Fig 4, areas highlighted with blue brackets).

### Initial determination of clinical sensitivity of each individual biomarker assay

Given that the ultimate goal of this work is to develop a diagnostic test for both New and Old World VL, we proceeded with a preliminary clinical validation of the six leishmanial biomarker detection assays using urine from patients with VL from Brazil (n = 24) and Kenya (n = 45). Urine was collected prior to initiating VL therapy. As controls, 25 urine samples were collected from healthy individuals from VL-endemic countries and 10 urine samples from healthy individuals from Massachusetts, USA. All urine samples were frozen (-80°C) prior to testing and handled identically upon thawing. The cutoff to discriminate positive from negative results was calculated as the average of the results for the control samples plus 3 SD. The standardization of the individual capture ELISA for each of the six markers was performed “blindly”, i.e., the test for each individual marker using each individual urine sample was performed without the knowledge of the results obtained for the other markers.

**Fig 6:**
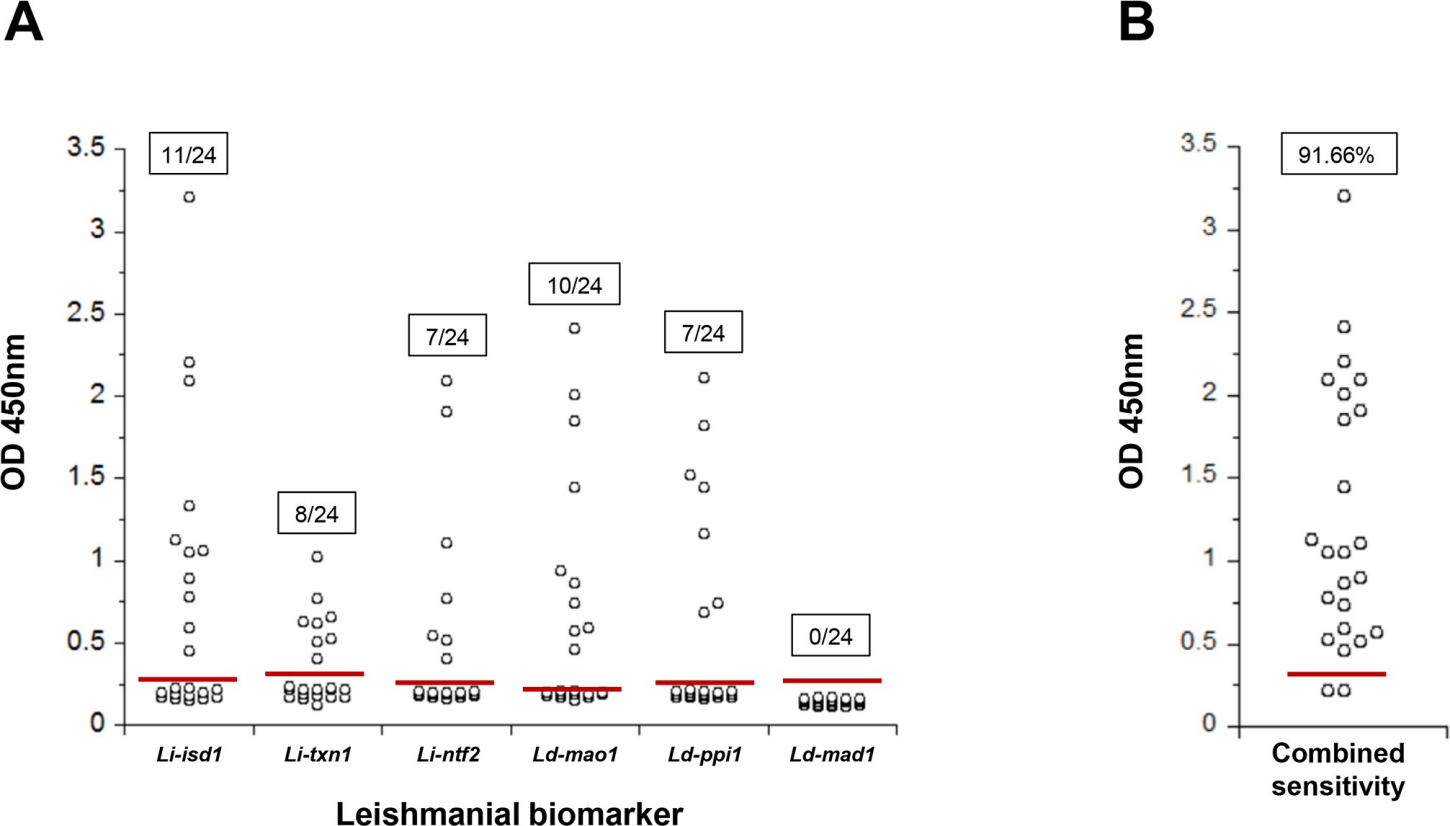
Capture ELISA for detection of *Li-isd1*, *Li-txn1*, *Li-ntf2*, *Ld-mao1*, *Ld-ppi1 and Ld-mad1* in urine of VL patients and local healthy controls from Brazil. ELISA was assembled as indicated in Fig 5. Urine samples were VL patients (n = 24) and healthy control subjects (n = 10). (A) Results represent the sensitivity of the assays performed to detect each individual biomarker. (B) Is a representation of the combined best results obtained for each of the urine samples that was positive (or not) with at least one of the individual biomarker assays. Red solid lines represent the cutoff values, which were calculated using the average of the OD obtained from the urine of normal healthy control subjects + 3 SD. These are representative results of at least three experiments performed at different times with the same urine samples and same capture ELISA.

The results showed that the sensitivities for the assays for *Li-isd1*, *Li-txn1*, *Li-ntf2*, *Ld-mao1*, *Ld-ppi1 and Ld-mad1* using urine samples from VL patients from Brazil were 45%, 33.3%, 29.1%, 41.6, 29.1% and 0% respectively (Fig 6).

**Fig 7:**
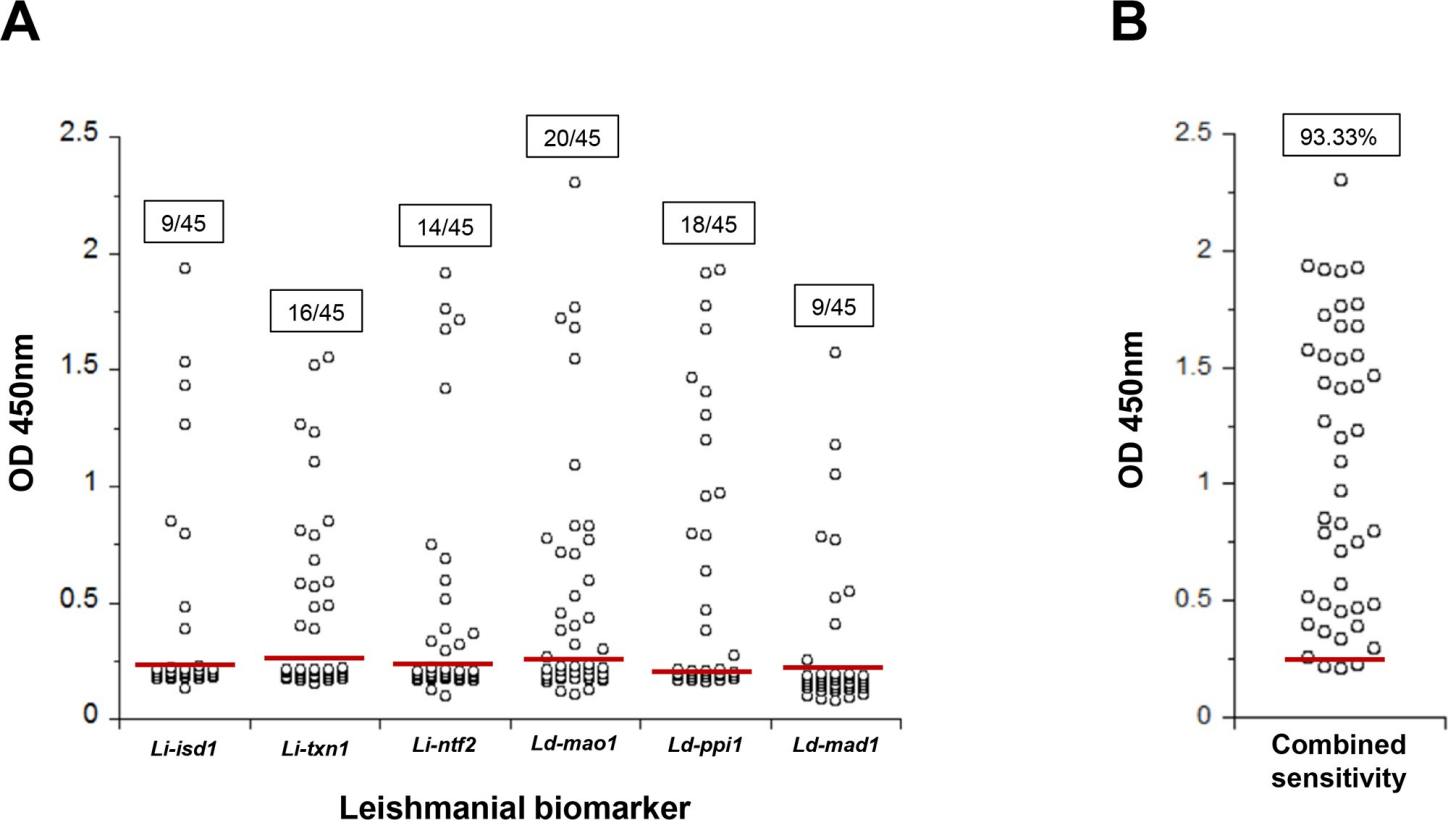
Capture ELISA for detection of *Li-isd1*, *Li-txn1*, *Li-ntf2*, *Ld-mao1*, *Ld-ppi1 and Ld-mad1* in urine of VL patients and local healthy controls from Kenya. ELISA was assembled as indicated in Fig 5. Urine samples were from VL patients (n = 45) and healthy control subjects (n = 24). (A) Results represent the sensitivity of the assays performed to detect each individual biomarker. (B) Is a representation of the combined best results obtained for each of the urine samples that was positive (or not) with at least one of the individual biomarker assays. Solid red lines represent the cutoff values, which were calculated using the average of the OD obtained from the urine of normal healthy control subjects + 3 SD. These are representative results of at least three experiments performed at different times with the same urine samples and same capture ELISA.

The sensitivities of the individual assays performed with VL patients from Kenya were 20%, 35.5%, 31%, 44.4%, 40% and 20% respectively (Fig 7).

Importantly, an in-depth analysis of the results of each individual assay revealed that 0–5 biomarkers could be detected in each patient’s urine sample from either Brazil or Kenya (Tables 3 and 4 respectively) and that the combined sensitivity of these markers is 91.66% and 93.33% for VL from these two countries respectively. At this time point, we did not attempt to correlate the results with disease severity because this evaluation is beyond the scope of the present study and would require a much larger patient sample size and a specific Human Subject Protocol approval, which would be different from the ones approved for the current study. Nonetheless, the results highlight and confirm our previous observation that a highly sensitive assay would need to include reagents that detect several different biomarkers preferentially assembled in a multiplexed format.

**Table 3:** Frequency of individual leishmanial biomarkers detected by capture ELISA in 24 urine samples obtained from VL patients from Brazil.

	# of detected markers per urine sample						
	None	1	2	3	4	5	6
# of urine samples	2	8	8	5	1	None	None

**Table 4:** Frequency of individual leishmanial biomarkers detected by capture ELISA in 45 urine samples obtained from VL patients from Kenya.

	# of detected markers per urine sample						
	None	1	2	3	4	5	6
# of urine samples	3	17	11	9	4	1	None

### Configuration and initial clinical validation of a multiplexed assay

A multiplexed assay offers several advantages over single assays; these include the ability to detect several biomarkers per test (which is crucial to achieving a high degree of sensitivity), the speed of performing the test and lower cost to produce and use compared to detecting individual biomarkers.

**Fig 8:**
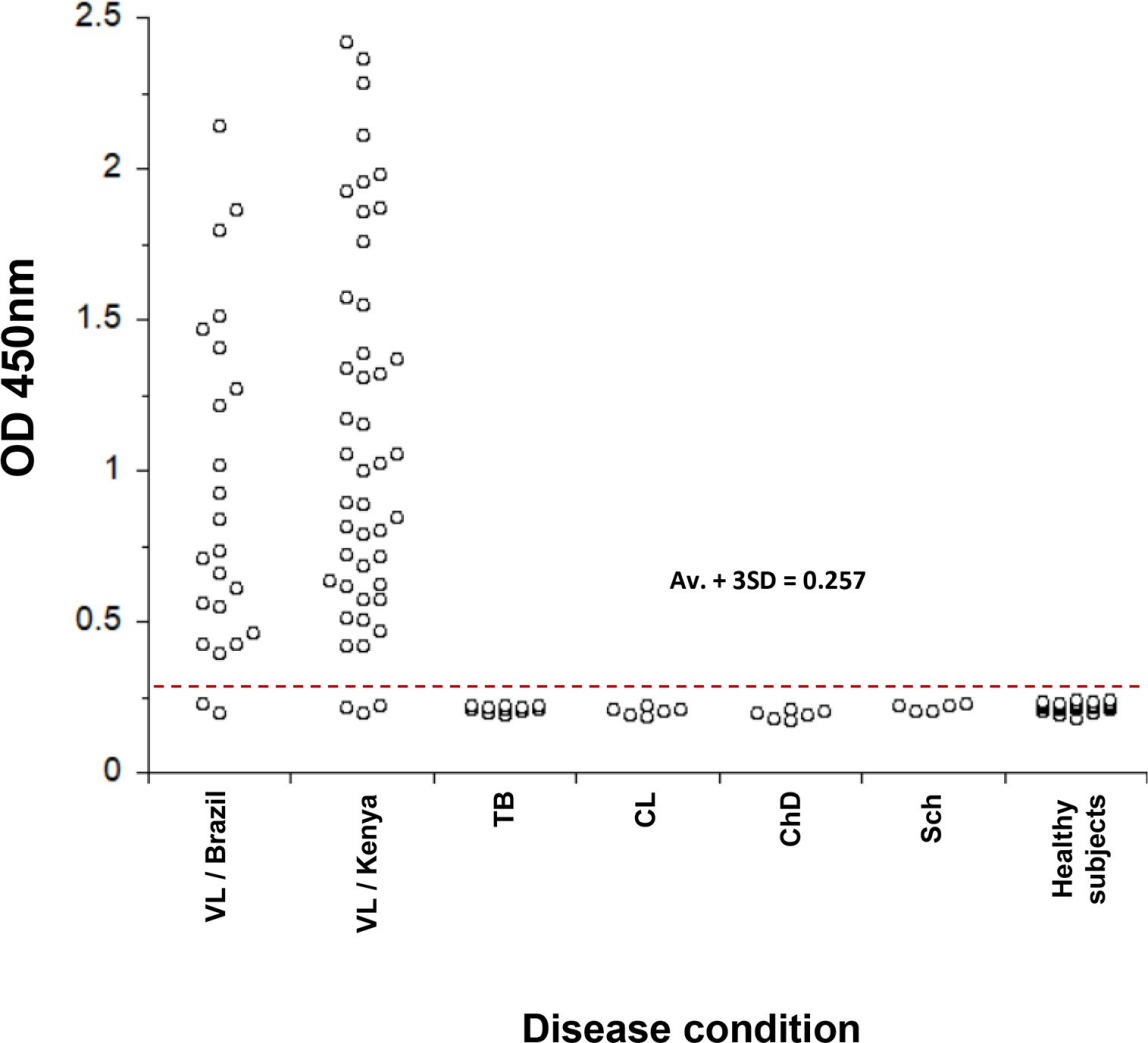
Initial clinical validation of a multiplexed assay for the diagnosis of VL from Brazil and from Kenya. ELISA plates were coated with a pool of affinity purified antibodies specific for all six biomarkers (*Li-isd1*, *Li-txn1*, *Li-ntf2*, *Ld-mao1*, *Ld-ppi1 and Ld-mad1*) followed by blocking and overnight incubation with urine samples from VL patients from Brazil (n = 24), VL patients from Kenya (n = 45) and urine samples from non-VL patients with the following diseases: CL (cutaneous leishmaniasis), n = 6; CD (Chagas disease), n = 6; Sch (schistosomiasis), n = 6; and TB (tuberculosis), n = 12. In addition, urine from 35 healthy control subjects was also included. Plates were washed and wells were incubated with a second pool containing biotinylated mAbs specific for the six leishmanial antigens. Wells were then incubated with streptavidin labeled peroxidase, the substrate H_2_O_2_ and the chromophore TMB. OD was then read at 450nm. The dashed red line represents the cutoff value (0.26) calculated as described in the legend of Fig 6. This is a representative result of at least three experiments performed at different times with the same urine samples.

The multiplexed ELISA designed to simultaneously detect *Li-isd1*, *Li-txn1*, *Li-ntf2*, *Ld-mao1*, *Ld-ppi1* and *Ld-mad1* was assembled using a pool of the six specific mAbs that were used in the individual assays (mAbP11, mAbP212, mAbP33, mAbP41, mAbP64 and mAbP713) as capture reagents. Similarly, the developing reagent consisted of a pool of the biotinylated mAbs used in the individual assays (mAbP115, mAbP218, mAbP311, mAbP49, mAbP618 and mAbP79). A preliminary evaluation of the multiplexed assay revealed no loss of sensitivity when compared to the individual assays (S1 Fig). To begin the clinical validation of the multiplexed assay we used the same urine samples from VL patients from Brazil and Kenya that were used to validate the individual capture ELISAs. In addition, several control samples from non-VL patients who had other infectious diseases (cutaneous leishmaniasis, n = 6; Chagas disease, n = 6; schistosomiasis, n = 6; and tuberculosis, n = 12) were also tested. The results are illustrated in Fig 8 and show that the multiplexed assay has excellent sensitivity for the diagnosis of VL from Brazil (91.66%) and Kenya (93.33%). Interestingly, these sensitivities were the same as those observed for the calculated combined individual assays with the same samples giving positive/negative results. Importantly, the multiplexed assay had a specificity of 100% since no positive result was observed with urine samples from healthy control subjects or from patients having other infectious diseases. Therefore, the multiplexed assay assembled with mAbs specific for *Li-isd1*, *Li-txn1*, *Li-ntf2*, *Ld-mao1*, *Ld-ppi1* and *Ld-mad1* confirms and expands our former proof-of-concept observations made with polyclonal antibodies.

## Discussion

Visceral leishmaniasis (VL) or kala-azar is a systemic parasitic disease that is endemic in 75 countries with more than 500 million people at risk of infection. It is estimated to affect 50,000–90,000 people each year, with 90% of the cases occurring in India, Kenya, Somalia, Sudan, South Sudan and Brazil [2]. VL is a fatal disease if not treated [1, 2]. Laboratory diagnosis of this disease can be performed using several different approaches. However, there is still a need for the development of a simple, non-expensive, sensitive and specific test that can be used worldwide not only for the accurate diagnosis of this serious disease but also to monitor the treatment efficacy [35].

In recent years, we have shown that protein antigens from the pathogens *L*. *infantum* and *L*. *donovani* could be detected in urine of patients with active VL using mass spectroscopy. The discovery of these pathogen proteins led us to the development of a urine-based capture ELISA for the diagnosis of this serious disease. This ELISA, which was assembled with polyclonal antibodies raised in rabbits and chickens, constitutes a successful proof of concept for the development of a urine-based diagnostic test for VL. A multiplexed assay based on five of the biomarkers discovered could be developed and shown to be highly sensitive and specific for the diagnosis of VL [23–25]. However, the use of polyclonal antibodies is a barrier to scaling-up such a test for commercial production. A proven alternative is the use of mAbs, which circumvents this limitation. Here, we report our results on the production and use of mAbs specific for the *L*. *donovani / L*. *infantum* antigens *Li-isd1*, *Li-txn1*, *Li-ntf2*, *Ld-mao1*, *Ld-ppi1 and Ld-mad1* for the development of a sensitive and specific multiplexed test for the diagnosis of VL.

Our initial efforts were concentrated on the validation of the biomarker *Ld-mad1* (putative *L*. *donovani* malate dehydrogenase) because producing both purified recombinant protein and specific rabbit and chicken antibodies to detect this molecule proved difficult in our former study. In the present study, we opted to produce monoclonal antibodies (mAbs) to *Ld-mad1* to circumvent the non-specific reactivity observed with the polyclonal antibodies generated against this molecule. The generation of the mAbs using a conventional procedure was readily achieved and 20 clones producing antibodies that were highly specific for *Ld-mad1* were selected using Western blot analysis. Specificity was determined by the detection of a strong band that matched the expected MW of *Ld-mad1*. This was easily obtained regardless of the purity of the recombinant protein used in the Western blot and to immunize the mice. Furthermore, the reactivity and specificity of the mAbs to the native *Ld-mad1* protein (in contrast to the recombinant molecule) was also determined by Western blot analysis using leishmania parasite cell extract. The *Leishmania* malate dehydrogenase protein was originally described as preferentially being produced by amastigote forms of *Leishmania mexicana* [33, 34]. Therefore, to validate the reactivity of the mAbs with native *Ld-mad1* we performed Western blot analysis using both amastigote and promastigote forms of *L*. *donovani*. The results clearly confirmed that *L*. *donovani* malate dehydrogenase (*Ld-mad1*), similarly to its production by *L*. *mexicana mexicana*, is preferentially or only expressed by *L*. *donovani* amastigotes.

The generation, selection, and purification of mAbs to all other five biomarkers were also obtained without difficulty. For practical reasons, only IgG producing clones were selected. Between 4–7 clones per biomarker were obtained. For each marker we were able to purify 15–20 mg of specific IgG from 3–7 clones, which were used for the selection of pairs of antibodies for assembling the capture ELISA to detect each biomarker. Interestingly and unexpectedly, the best antibody pairs for each of all six biomarkers recognized linear epitopes that were almost contiguous to each other in opposition to distantly located epitopes in the amino acid sequence of each molecule. At this point, we do not have an explanation for this observation, but it is possible that the binding of an antibody to a given linear epitope will partially change the tertiary structure of the protein, which might result in the exposure of contiguous but not distant linear epitopes of the molecule. Although an interesting observation, we did not explore it any further because it is beyond the scope of this study.

The ultimate goal of this work was to translate our preliminary proof of concept observation, i.e., the development of an antigen detection diagnostic test for VL using polyclonal rabbit IgG and chicken IgY, into a test that instead uses immortalized mAbs specific for each of six leishmanial biomarkers that we have previously described. This former work, demonstrated the proof of concept that an antigen detection test could be developed for the diagnosis of VL. Unfortunately, because that study was performed with polyclonal antibodies the results could not be directly translated into a de facto clinical test for VL. In contrast, the current work presents data that supports the immediate translation of the results into a new clinically useful diagnostic test for VL. Immortalized mAbs, in contrast to polyclonal antibodies, are suitable reagents for mass scale-up and continuous production of the final test. Initial validation of the ELISAs assembled to detect each one of the six biomarkers confirmed that the selected mAb pairs had an excellent biochemical sensitivity 15–45 pg/ml. Importantly, urine causes little or no interference in the assay sensitivity when compared to assays performed with the biomarkers diluted in PBS/BSA (S2 Fig). The clinical sensitivity of each assay confirmed our previous observations, that no single marker is sufficient for the development of a sensitive assay. Indeed, with the exception of the assay to detect *Ld-mad1*, the individual sensitivity varied from 20% to 50% of the VL patients from either Brazil or Kenya. In contrast, the assay to detect *Ld-mad1* was negative with all urine samples from VL from Brazil but had a sensitivity of 20% among the urine samples from VL patients from Kenya. It is possible that this lower sensitivity to detect *Ld-mad1* is due to the fact that this molecule has a higher MW than the other biomarkers, which may limit its excretion in the urine as a full-length protein, thus complicating its detection by the capture ELISA. Also intriguing was that fact that *Ld-mad1* was not detected in any of the 24 urine samples from VL patients from Brazil. At this point we do not have a clear explanation for this results. However, it is possible that it could be due to the limited number of samples that was thus far used for this validation. It is also theoretically possible that *Ld-mad1* is not eliminated in the urine of patients of VL caused by *L*. *infantum*.

Nonetheless, because many urine samples were positive for several of the six markers (Tables 3 and 4) it resulted in a combined sensitivity of 91.66% and 93.33% for the Brazil and Kenya urine samples respectively. These results concur with our previous observations indicating that a highly sensitive test requires the simultaneous detection of several VL biomarkers, either separately or in a multiplex format [23].

Indeed, the mAb-based multiplexed assay assembled to detect all six markers simultaneously showed excellent sensitivity and specificity. The overall sensitivity was again 91.66% for VL from Brazil and 93.33% for VL from Kenya, which confirms the calculated combined sensitivity of the individual assays and validates the multiplexed assay. Importantly, the sensitivity of this urine-based test is much higher than that observed for the invasive tests that detect the parasites themselves (~50%) and similar to that of methods that detect parasite nucleic acids, which is approximately 90% [4, 11, 36]. Moreover, the overall specificity of the multiplexed test was 100% when using urine samples not only from healthy control subjects but from non-VL patients who have other infectious diseases (cutaneous leishmaniasis, Chagas disease, schistosomiasis, and tuberculosis). We recognize that the sensitivity/specificity of the assembled multiplexed assay thus far is based on a limited number of urine samples from both patients and controls. Nonetheless, these results are very encouraging. We are in the process of expanding the clinical validation of this promising new test for the diagnosis of VL in order to translate it into a clinical tool. This validation will include a much larger sample size and will have patients not only from Brazil and Kenya but from other endemic countries as well, such as India and Bangladesh. In addition, we are evaluating the suitability of the mAbs developed for assembly into a reliable immunochromatographic rapid test for point-of-care diagnosis of VL.

Finally, because the multiplexed assay described here is assembled with highly defined and specific mAbs, this test by definition, is much more accurate and specific than assays assembled with polyclonal antibodies. Therefore, the new mAb-based multiplexed assay described in this communication will be of great utility not only for the diagnosis of active VL but also as an important tool to monitor the treatment efficacy for this disease. In fact, we have preliminary proof-of-concept evidence for this possible latter use from an ELISA assembled with conventional rabbit and chicken antibodies [25]. Moreover, the new mAb-based multiplexed assay should be a useful resource for diagnosis of other clinical forms and/or the severity of the disease e.g., asymptomatic VL, post kala-azar dermal leishmaniasis, VL/HIV co-infection, etc.

## Supporting information

S1 Fig Sensitivity of a multiplexed capture ELISA assembled to detect six *Leishmania* biomarkers (antigens) compared to capture ELISAs assembled to individually detect each biomarker. For single antigen detection capture ELISA (A), plates were coated with a single mAb antibody specific for one of the following leishmanial biomarkers: iron superoxide dismutase 1 (*Li-isd1*), tryparedoxin 1 (*Li- trx1*), nuclear transport factor 2 (*Li-ntf2*), maoC dehydralase (*Ld-mao1*), peptidyl-prolyl cis-trans isomerase (*Ld-ppi1*) and malate dehydrogenase (*Ld-mad1*) an individual biomarker. For multiplexed capture ELISA (B) plates were coated with a pool containing mAbs specific for the six biomarkers. For the single assay, detection was performed using a single biotinylated mAb specific for a different epitope than that recognized by the mAb used to coat the plates. For the multiplexed assay, detection was performed using a pool containing biotinylated mAbs that were specific for different epitopes of each individual antigen that were recognized by the pool of mAbs used to coat the plates. Reactions were developed after addition of peroxidase streptavidin A plus the substrate H_2_O_2_ and the chromophore TMB. Results are expressed as OD read at 450nm. Note that there is no loss of sensitivity of the multiplexed assay compared to the single assay.
(PDF)

S2 Fig Sensitivity of a multiplexed capture ELISA for detection of leishmanial biomarkers (antigens), spiked in either buffer or in urine of healthy subjects. A pool, containing mAbs specific for the markers *Li-isd1*, *Li-txn1*, *Li-ntf2*, *Ld-mao1*, *Ld-ppi1*, and *Ld-mad1*, each at 2 μg/ml was used to coat the ELISA plates. Wells were then incubated with various concentrations of the six makers diluted either in PBS plus 1% BSA or in urine from healthy subjects. Detection was performed using a pool of biotinylated mAbs that were specific for different epitopes of each individual marker that were recognized by the pool of mAbs used to coat the plates. Reactions were developed after addition of peroxidase streptavidin A plus the substrate H_2_O_2_ and the chromophore TMB. Results are expressed as OD read at 450nm. Note that urine does not interfere with the sensitivity of the multiplexed assay.
(PDF)
