# Supplementary figures and images for: Urine-based antigen detection assay for diagnosis of visceral leishmaniasis using monoclonal antibodies specific for six protein biomarkers of Leishmania infantum / Leishmania donovani
Source: PLoS Negl Trop Dis. 2020 Apr 23;14(4):e0008246. doi: 10.1371/journal.pntd.0008246 (PMC7200010; doi:10.1371/journal.pntd.0008246)

**A**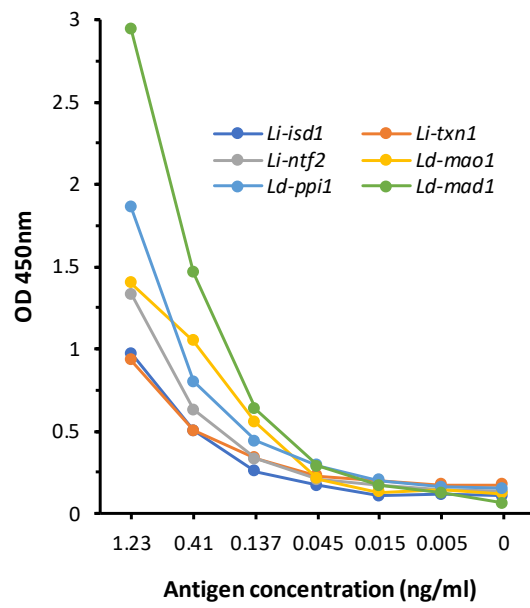**B**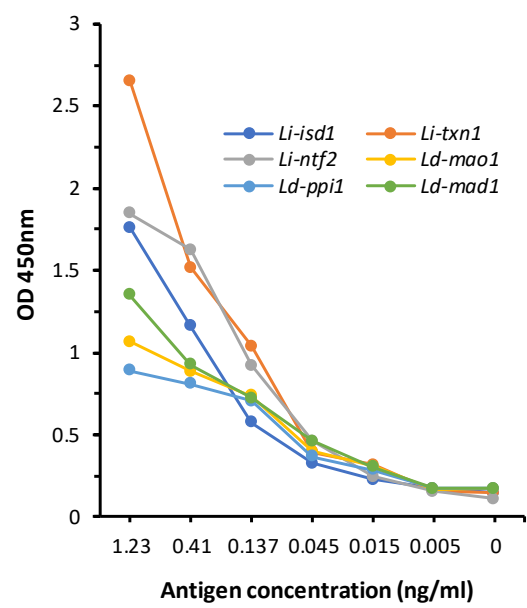

Supplement: S1 Fig — For single antigen detection capture ELISA (A), plates were coated with a single mAb antibody specific for one of the following leishmanial biomarkers: iron superoxide dismutase 1 (Li-isd1), tryparedoxin 1 (Li- trx1), nuclear transport factor 2 (Li-ntf2), maoC dehydralase (Ld-mao1), peptidyl-prolyl cis-trans isomerase (Ld-ppi1) and malate dehydrogenase (Ld-mad1) an individual biomarker. For multiplexed capture ELISA (B) plates were coated with a pool containing mAbs specific for the six biomarkers. For the single assay, detection was performed using a single biotinylated mAb specific for a different epitope than that recognized by the mAb used to coat the plates. For the multiplexed assay, detection was performed using a pool containing biotinylated mAbs that were specific for different epitopes of each individual antigen that were recognized by the pool of mAbs used to coat the plates. Reactions were developed after addition of peroxidase streptavidin A plus the substrate H2O2 and the chromophore TMB. Results are expressed as OD read at 450nm. Note that there is no loss of sensitivity of the multiplexed assay compared to the single assay. (PDF) [file pntd.0008246.s001.pdf]

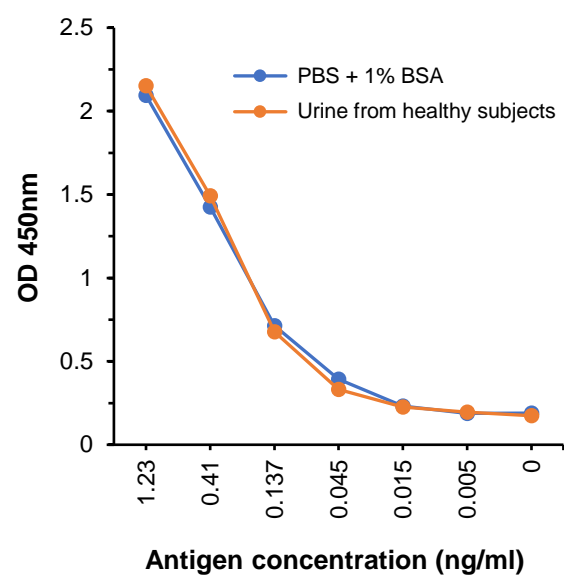

Supplement: S2 Fig — A pool, containing mAbs specific for the markers Li-isd1, Li-txn1, Li-ntf2, Ld-mao1, Ld-ppi1, and Ld-mad1, each at 2 μg/ml was used to coat the ELISA plates. Wells were then incubated with various concentrations of the six makers diluted either in PBS plus 1% BSA or in urine from healthy subjects. Detection was performed using a pool of biotinylated mAbs that were specific for different epitopes of each individual marker that were recognized by the pool of mAbs used to coat the plates. Reactions were developed after addition of peroxidase streptavidin A plus the substrate H2O2 and the chromophore TMB. Results are expressed as OD read at 450nm. Note that urine does not interfere with the sensitivity of the multiplexed assay. (PDF) [file pntd.0008246.s002.pdf]
